# Lumbopelvic Complex Alignment Defects in Adolescents: Relationships with Temperament and Implications for Individualised Prevention and Rehabilitation Strategies

**DOI:** 10.3390/jcm15103937

**Published:** 2026-05-20

**Authors:** Jacek Wilczyński, Małgorzata Gawlik, Kamil Margiel, Paulina Szumilas, Katarzyna Bieniek, Jakub Bąk, Marta Mierzwa-Molenda

**Affiliations:** Collegium Medicum, Jan Kochanowski University in Kielce, 25-317 Kielce, Poland; gawlik.malgosia@wp.pl (M.G.); kamilo48@onet.pl (K.M.); palulinal@gmail.com (P.S.); katarzyna.bieniek@onet.com.pl (K.B.); bak.jakub96@gmail.com (J.B.); martamierzwa@wp.pl (M.M.-M.)

**Keywords:** body posture disorders, lumbopelvic complex, temperament, pelvic torsion DL–DR, rasterstereography, prevention, rehabilitation

## Abstract

**Background/Objectives:** The lumbopelvic complex plays a key role in postural regulation, core stabilisation and biomechanical coordination of spinal and pelvic alignment. The aim of the study was to analyse relationships between lumbopelvic complex alignment defects and temperament types in adolescents, and to evaluate their potential relevance for more individualised prevention and rehabilitation strategies. **Methods:** The study is of a cross-sectional design. The research included 273 adolescents aged 16–17 years. Body posture was assessed using the DIERS Formetric III 4D system based on rasterstereography. Temperament traits were evaluated using the Pavlovian Temperament Survey (PTS). **Results:** Body posture in the examined adolescents was characterised by high variability, and the majority of participants presented abnormalities in lumbopelvic alignment and spinal curvatures. The most common finding was flattening of physiological spinal curvatures, particularly reduced thoracic kyphosis coexisting with normal or reduced lumbar lordosis. No significant associations were found between temperament and most of the analysed postural parameters. Significant differences were identified only for the pelvic torsion DL–DR parameter (H = 9.96; *p* = 0.019). Pelvic torsion DL–DR values were significantly higher in the sanguine temperament group compared to the phlegmatic (*p* = 0.013) and choleric groups (*p* = 0.015). Higher values of this parameter were also observed in adolescents with a melancholic temperament. **Conclusions:** Temperament may selectively influence mechanisms underlying lumbopelvic complex control, particularly regarding the pelvic torsion DL–DR parameter. Incorporating temperamental characteristics into postural diagnostics may support the development of more individualised prevention and rehabilitation programmes aimed at improving core stabilisation, motor control and functional control of the lumbopelvic complex in adolescents.

## 1. Introduction

Body posture is a complex neurophysiological and biomechanical process resulting from the integration of sensory mechanisms, central nervous system activity and musculoskeletal system function. Proper postural organisation enables the maintenance of body stability with minimal energy expenditure and provides optimal conditions for musculoskeletal function [1,2]. Contemporary posturology considers posture as a dynamic regulatory system dependent on the interaction of biomechanical, neurophysiological, sensory and behavioural factors rather than merely static alignment of skeletal structures. A key role in postural organisation is attributed to the lumbopelvic complex, which constitutes a central functional unit that integrates the spine, pelvis, and lower extremities into a coordinated biomechanical chain. This complex is responsible for core stabilisation, trunk alignment control, and the appropriate distribution of loads acting on the musculoskeletal system during standing, gait, and other functional activities. The proper function of the lumbopelvic complex depends on effective cooperation between sensory-motor mechanisms and appropriate activation of trunk stabilising muscles. Disturbances in lumbopelvic control may lead to compensatory postural patterns and asymmetries in pelvic and spinal alignment. Increasing attention in postural diagnostics has been directed towards spatial pelvic alignment parameters, including pelvic obliquity, pelvic tilt and torsion [3,4]. Particular importance is attributed to the pelvic torsion DL–DR parameter, which is considered a sensitive indicator of functional disturbances within the lumbopelvic complex. This parameter describes the mutual alignment of both sides of the pelvis and may reflect compensatory mechanisms related to muscle tension regulation and neuromuscular control [5,6]. Alterations in pelvic torsion DL–DR may result not only from biomechanical factors, but also from individual regulatory properties of the nervous system [7,8]. One biological factor potentially influencing postural organisation is temperament, understood as a relatively stable set of traits associated with the properties of excitation, inhibition and mobility of nervous processes [9,10]. Contemporary psychophysiological models suggest that temperament may modulate emotional reactivity, psychomotor activity, muscle tension regulation and postural adaptation strategies. Individuals representing different temperament types may therefore demonstrate distinct patterns of neuromuscular control and postural stabilisation [11,12,13]. Adolescence is a particularly important period for postural development due to intensive somatic, hormonal and neuromotor changes. During this stage, individual movement control and postural stabilisation strategies become consolidated. At the same time, the contemporary lifestyle of adolescents, characterised by reduced physical activity and prolonged sitting, contributes to disturbances in lumbopelvic alignment and spinal curvatures. Despite the growing interest in the neurophysiological determinants of posture, studies investigating relationships between temperament and lumbopelvic complex control remain limited [14]. Previous research has been mainly focused on global postural parameters or conventional assessment of spinal curvatures, whereas spatial pelvic alignment, including the pelvic torsion DL–DR parameter, has been analysed less frequently [15]. Moreover, the majority of earlier studies have relied on clinical or two-dimensional postural assessment methods, which do not allow comprehensive evaluation of spatial asymmetries and subtle pelvic alignment disturbances. The innovative aspect of the present research lies in combining the analysis of temperamental traits with three-dimensional postural assessment using the DIERS Formetric III 4D system based on rasterstereography. This method enables a non-invasive, precise, and objective evaluation of spinal and pelvic alignment, without exposure to ionising radiation. Of particular importance is the application of the pelvic torsion DL–DR parameter as a potential indicator of neurophysiological mechanisms involved in postural regulation associated with nervous system properties [15]. The study also involves adolescents during a period of intensive biological and functional development, which further increases the scientific value of the obtained results. The aim of the present research was to analyse relationships between lumbopelvic complex alignment defects and temperament types in adolescents aged 16–17 years. Particular attention was devoted to the assessment of the pelvic torsion DL–DR parameter as a potential indicator of postural regulatory mechanisms associated with nervous system properties.

## 2. Materials and Methods

### 2.1. Participant Characteristics

The study included 273 secondary school students aged 16–17 years. It was conducted at the Posturology Laboratory of Collegium Medicum, Jan Kochanowski University in Kielce, on the following dates: 21 March 2023–23 March 2023. The study was of a cross-sectional design. The sample size of 273 school-age children was determined to provide adequate statistical power for detecting significant differences in postural parameters between temperament groups. Assuming the prevalence of postural disorders in the school-age population is approximately 25–35%, a sample size exceeding 250 participants would provide an estimated margin of error of approximately ±5% at a 95% confidence level, which is consistent with standard requirements for epidemiological studies. Prior to participation, both students and their parents or legal guardians were informed of the aim and procedures of the study, as well as their right to withdraw at any time. Participation required obtaining written informed consent from students and their parents or legal guardians. The study was conducted in conditions ensuring participants’ psychological comfort. Ethical approval was obtained from the Bioethics Committee of Collegium Medicum, Jan Kochanowski University in Kielce (No. 55/29.07.2021). The inclusion criteria were as follows: age 16–17 years; attending secondary school; absence of diagnosed congenital musculoskeletal disorders or central nervous system diseases that could affect psychomotor development; lack of physical or intellectual disability; and written informed consent for participation from participants’ parents or legal guardians.

### 2.2. Methods and Research Instruments

#### 2.2.1. Body Composition Assessment

Body composition assessment was performed using the TANITA MC-780 bioelectrical impedance analyser (Tokyo, Japan), enabling multi-frequency body composition analysis based on the bioelectrical impedance analysis (BIA) method. The examination was conducted in a standing position, barefoot, using electrodes located on the platform and in the device handles. The measurement time was approximately 20–30 s. The following parameters were analysed: body mass (mass, kg); body mass index (BMI, kg/m^2^); percentage of body fat (FATP, %); fat mass (FATM, kg); predicted muscle mass (PMM, kg); bone mineral mass (BONEM, kg); fat-free mass (FFM, kg); total body water (TBW, kg); basal metabolic rate (BMR, kJ); and phase angle (PA, °). The BIA method is based on the assessment of electrical conductivity in body tissues. Muscle tissue, containing large amounts of water and electrolytes, conducts electrical current more easily than adipose tissue, which demonstrates greater electrical resistance. Based on the obtained impedance values, the device calculates body composition parameters using appropriate mathematical algorithms.

#### 2.2.2. Body Posture Assessment

Body posture was assessed using an optoelectronic method with the DIERS Formetric III 4D system, enabling three-dimensional analysis of the back surface based on rasterstereography. The method involves projecting horizontal light stripes onto the back surface of the subject, with their deformation recorded by a digital camera. Based on the obtained data, a precise three-dimensional model of the back surface is created, allowing assessment of the spatial alignment of the spine, trunk, and pelvis [16]. The DIERS Formetric III 4D method is a contactless, automatic, and non-invasive technique for body posture assessment that does not require exposure to ionising radiation. Unlike conventional radiographic examination, it allows repeated follow-up assessments, without exposing the participant to X-rays. The reliability and repeatability of the measurements have been confirmed through comparison with digital radiographic analysis of the spine. The examination was performed in a dimly lit room to eliminate the influence of external light on measurement quality. The participants stood in a habitual standing position at a distance of approximately 2–3 metres from the device [17]. During the examination, the participants wore only shorts, and reflective elements such as jewellery and metallic accessories were removed before measurement. The analysis was performed using DiCam v2.5.15 software in the ‘Average’ mode, which involves recording a series of twelve consecutive images that are subsequently averaged to increase measurement accuracy and repeatability. The analysis included selected parameters describing body posture in the sagittal, frontal and transverse planes. Vertical deviation VP-DM was assessed both in degrees (vertical deviation, VP–DM [°]) and millimetres (vertical deviation, VP–DM [mm]). This parameter determines trunk axis deviation from the vertical line between the vertebra prominens (VP) and the midpoint between the lumbar dimples (DM), representing an indicator of trunk asymmetry. Another analysed parameter was pelvic obliquity DL–DR expressed in degrees (pelvic obliquity, DL–DR [°]) and millimetres (pelvic obliquity, DL–DR [mm]) [18]. This parameter defines the height difference between the right and left lumbar dimples relative to the horizontal plane and enables assessment of pelvic asymmetry in the frontal plane. Positive values indicate a higher position of the right side of the pelvis, whereas negative values represent a higher position of the left side. Pelvic torsion DL–DR (pelvic torsion, DL–DR [°]), describing rotational alignment of the pelvis at the lumbar dimples, was also analysed. This parameter reflects reciprocal pelvic torsion and enables assessment of alignment disturbances in the transverse plane. Pelvic tilt at the lumbar dimples (pelvic tilt (dimples) [°]) was also assessed as an indicator of pelvic alignment in the sagittal plane. This parameter is calculated as the average inclination of the plane determined by the DL and DR points. In the sagittal plane, the kyphosis angle (kyphosis angle [°]), describing the size of physiological posterior curvature in the thoracic spine, and the lordosis angle (lordosis angle [°]), describing the degree of anterior curvature in the lumbar spine, were analysed [19]. These parameters constitute the basic indicators used for the assessment of physiological spinal curvatures and enable the determination of their flattening or deepening. In the transverse plane, surface rotation (surface rotation (rms) [°]) was evaluated as the mean value of back surface rotation along the spinal symmetry line. This parameter enables assessment of rotational components of trunk deformities characteristic, among others, of functional and structural scoliosis [20]. Trunk torsion (trunk torsion [°]), describing global trunk rotation relative to the body’s vertical axis, was also analysed. This parameter reflects asymmetry in the spatial alignment of the shoulder and pelvic girdles. The final assessed parameter was lateral deviation VP–DM (lateral deviation, VP–DM (rms) [mm]), representing the root mean square value of lateral deviation of the spinal spinous process line from the body symmetry axis in the frontal plane. This parameter enables assessment regarding the size of lateral spinal asymmetries and the degree of spinal deviation from the central body axis [21].

#### 2.2.3. Assessment of Temperament Type

Temperament traits were assessed using the Pavlovian Temperament Survey (PTS), developed by Jan Strelau, Alois Angleitner, and Bogdan Zawadzki. The instrument was implemented to diagnose behavioural characteristics concerning the basic properties of the nervous system in accordance with the concept of nervous system types. The PTS questionnaire is considered one of the most significant Polish psychometric instruments used in temperament research and the only Polish temperament questionnaire that has achieved broad international application. The theoretical foundations of the questionnaire derive from Pavlov’s concept of higher nervous activity. Pavlov regarded the type of nervous system as the physiological basis of temperament, primarily related to the organism’s ability to adapt to changing environmental conditions. In this model, particular importance is attributed to the properties of excitation and inhibition processes as well as their mutual relationships [9]. The PTS questionnaire enables assessment of three fundamental properties of the nervous system: strength of excitation (SE), strength of inhibition (SI), and mobility of nervous processes (MNP). Strength of excitation refers to the functional capacity of the central nervous system and is expressed as the ability to respond adequately to strong or prolonged stimuli without the occurrence of protective inhibition. This characteristic is associated with emotional resilience, psychological endurance and tolerance to stress and overload. Strength of inhibition refers to the ability to control and suppress responses according to situational demands. It is reflected in the capacity to delay, interrupt or extinguish specific behaviours, and corresponds to the psychological dimension of self-control and behavioural regulation. A high level of this trait is associated with better emotional control and more effective social adaptation. Mobility of nervous processes refers to the ability to rapidly change reactions in response to changing environmental conditions. This characteristic reflects the flexibility of central nervous system functioning, adaptability, and the ease of transition from excitation to inhibition, and vice versa. From a psychological perspective, it corresponds to the ability to modify behaviour according to current situational demands. Based on the configuration of these nervous system properties, four classical temperament types are distinguished: sanguine, phlegmatic, choleric, and melancholic. The sanguine temperament is characterised by a strong, balanced and mobile nervous system, associated with high psychological resilience and adaptive abilities. The phlegmatic temperament is characterised by a strong and balanced but less mobile nervous system, resulting in greater behavioural stability but lower flexibility in reactions. The choleric temperament is associated with a predominance of excitation processes over inhibition processes, which may manifest as increased impulsivity and emotional reactivity. In contrast, the melancholic temperament corresponds to a weak type of nervous system characterised by high sensitivity to stimuli and lower resistance to psychological overload. The PTS questionnaire consists of 57 statements rated on a four-point response scale: 1—strongly agree; 2—rather agree; 3—rather disagree; and 4—strongly disagree. The results are calculated by summing the scores obtained for individual scales and subsequently interpreting them according to sten norms. Higher scores indicate greater intensity of a given temperament trait. The questionnaire is designed for individuals aged 15–80 years and may be administered individually or in groups. The examination procedure followed standard questionnaire-based diagnostic procedures. Prior to the assessment, participants were informed about the aim and course of the procedure and subsequently provided informed consent for participation. The questionnaire completion time was not limited, and the completeness of responses was verified after completion. Interpretation of the results included both a psychometric aspect, relating the participant’s score to the normative group, and a psychological aspect, considering the functional significance of individual temperament traits in behavioural regulation, emotional resilience and adaptation to environmental conditions [10].

#### 2.2.4. Statistical Analysis

In order to verify the research questions and hypotheses, statistical analyses were performed using the IBM SPSS Statistics 25 package (IBM Corp., Armonk, NY, USA). Basic descriptive statistics were calculated for all of the analysed variables. The normality of data distribution was assessed using the Shapiro–Wilk test. Associations between categorical variables, including posture types and temperament types, were analysed using the χ^2^ test of independence. In cases where expected cell counts were insufficient, Fisher’s Exact test was applied. The strength of associations was additionally evaluated using Cramér’s V coefficient. Due to unequal group sizes and non-normal distribution of morphometric parameters, differences between temperament groups were analysed using the Kruskal–Wallis non-parametric analysis of variance (ANOVA). When statistically significant differences were identified, post hoc pairwise comparisons were performed using Dunn–Šidák tests. The results were presented as means (M), standard deviations (SDs), medians (Mes), and interquartile ranges (IQRs), depending on the type and distribution of data. The classical threshold of α = 0.05 was considered the level of statistical significance [22].

## 3. Results

In the present study, 273 students—125 female (45.8%) and 148 male (54.2%), aged 16 to 17 years (*M* = 16.51; *SD* = 0.50)—took part in the analysis. Basic descriptive statistics for all morphometric parameters are presented in Table 1. Shapiro–Wilk tests were statistically significant for most of the analysed variables, although skewness was relatively low, within the range of ±2, meaning that parametric tests could be performed in subsequent analyses if other assumptions were met.

**Table 2:** Distribution of temperament types among students with normal and abnormal body posture.

	Melancholic		Choleric		Sanguine		Phlegmatic		
Variable	*N*	%	*N*	%	*N*	%	*N*	%	Total
Normal	21	32.8%	2	3.1%	19	29.7%	22	34.4%	64
Abnormal	86	41.1%	20	9.6%	50	23.9%	53	25.4%	209
Total	107		22		69		75		273

Similar analyses were performed for three levels of both lordosis and kyphosis—reduced, normal and increased. However, no statistically significant result was observed: in lordosis: χ^2^(6) = 1.87; *p* = 0.932; *V* = 0.06; in kyphosis: χ^2^(6) = 4.93; *p* = 0.553; *V* = 0.10. The results are presented in Table 3.

**Figure 1:**
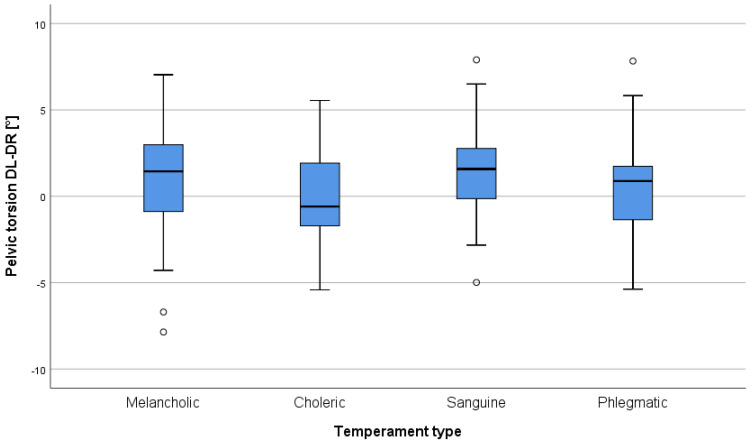
Box-and-whisker plot presenting Pelvic torsion parameter level across four temperament-type groups.

Furthermore, the Kruskal–Wallis non-parametric ANOVA was performed due to highly unequal group sizes in order to verify differences in morphometric parameters between temperament types. One statistically significant result was found for the pelvic torsion DL–DR parameter (Table 4). Therefore, post hoc analysis using Dunn–Šidák tests was conducted. Two statistically significant differences were identified. Values of the pelvic torsion DL–DR parameter were significantly higher in the sanguine temperament group compared to the phlegmatic (*p* = 0.013) and the choleric group (*p* = 0.015). Additionally, differences between the melancholic group and both the phlegmatic (*p* = 0.066) and choleric groups (*p* = 0.051) approached the level of statistical significance. Visual interpretation of Figure 1 indicates relatively high median values in both the melancholic and sanguine groups. No other differences were statistically significant. The results for the pelvic torsion DL–DR parameter are presented in Figure 1.

## 4. Discussion

The results of the present study demonstrate that relationships between lumbopelvic complex alignment defects and temperament in adolescents were limited and selective in nature. No statistically significant associations were found between temperament and most analysed postural parameters [23]. The only parameter for which a significant relationship was identified was pelvic torsion DL–DR. This observation may suggest that neurophysiological characteristics associated with temperament partially coexist with mechanisms regulating pelvic alignment; however, these relationships appear moderate and should not be interpreted as direct causal associations. Additionally, the obtained results confirm a high prevalence of postural defects in the examined group of adolescents. The majority of participants presented abnormalities in lumbopelvic alignment and physiological spinal curvatures, with the most common pattern being reduced thoracic kyphosis alongside normal or decreased lumbar lordosis. These findings are consistent with the currently observed increase in postural disorders among adolescents and may reflect the influence of sedentary lifestyle, reduced physical activity, and chronic static overload [24,25,26]. The lumbopelvic complex constitutes a central functional component of the postural system, integrating the spine, pelvis and lower extremities into a coordinated kinetic chain. Contemporary concepts of posturology indicate that pelvic alignment reflects not only biomechanical loading but also the organisation of neuromuscular control and adaptive processes. Consequently, pelvic torsion and asymmetry may result from the interaction of biomechanical, sensory, and neurophysiological factors rather than solely from structural changes.

The most important finding of the study concerned significant differences in the pelvic torsion DL–DR parameter between temperament groups [27,28]. Higher values of pelvic torsion were particularly observed in adolescents representing sanguine and melancholic temperament types, whereas lower values characterised individuals with phlegmatic and choleric temperaments. However, these differences were moderate, and some comparisons demonstrated only a trend towards statistical significance [29,30]. Interpretation of these findings may refer to the neurophysiological properties of the nervous system described in contemporary psychophysiological models of temperament. Melancholic temperament, associated with greater emotional reactivity and lower resistance to prolonged stimulation, may coexist with increased muscle tension reactivity and reduced stability of postural regulation mechanisms. In contrast, sanguine temperament, characterised by greater mobility of nervous processes, may favour greater variability of postural strategies and more dynamic neuromuscular adaptations [31,32]. Nevertheless, these interpretations remain hypothetical and require further confirmation through studies involving direct assessment of neurophysiological mechanisms and motor control. The obtained findings suggest that lumbopelvic alignment defects may partially reflect individual differences in the organisation of neuromuscular control. At the same time, postural alignment should be considered a multifactorial phenomenon dependent on biomechanical, developmental, environmental, and behavioural determinants [33,34]. Temperament may therefore represent one of the factors modulating postural regulation rather than a determinant of posture itself.

From a clinical perspective, the observed relationships may support the development of more individualised prevention and rehabilitation strategies for adolescents [35,36]. Considering temperamental characteristics during postural assessment may facilitate better adjustment of therapeutic interventions, particularly regarding muscle tension regulation, core stabilisation, motor control and adaptation to functional loading. Individuals characterised by greater emotional reactivity may require greater emphasis on techniques that improve muscle relaxation, breathing control, and psychophysiological regulation. In turn, individuals with greater mobility of nervous processes may benefit more from exercises focused on dynamic postural control and segmental stabilisation.

A major strength of the study was the use of objective three-dimensional postural assessment with the DIERS Formetric III 4D system, enabling precise analysis of spinal and pelvic alignment without exposing participants to ionising radiation. Another important advantage was the interdisciplinary approach combining biomechanics, posturology, and the psychophysiology of temperament.

The main limitation of the study was its cross-sectional design, which precluded causal inferences. Some temperament subgroups, particularly the choleric group, were relatively small, which may have reduced the statistical power of certain analyses. The temperament assessment was based on a questionnaire method and may thus have been influenced by the subjective nature of responses. Additionally, the analysis did not include potentially significant factors affecting postural regulation, such as physical activity, stress, breathing patterns or deep muscle function.

In conclusion, the present study supports the contemporary understanding of posture as a multidimensional neurophysiological and behavioural phenomenon rather than merely a static alignment of skeletal structures. The observed relationships between pelvic torsion and temperament suggest that certain features of lumbopelvic alignment may partially coexist with individual characteristics of nervous system functioning [11,12,13]. Such an integrative perspective may contribute to the development of more comprehensive diagnostic, preventive, and rehabilitation models for adolescents with postural alignment defects [37,38].

## 5. Conclusions

Body posture in adolescents aged 16–17 years was characterised by high variability, and the majority of the participants demonstrated abnormalities in lumbopelvic complex alignment and spinal curvatures. The most common finding was flattening of physiological spinal curvatures, particularly reduced thoracic kyphosis coexisting with normal or decreased lumbar lordosis, which may indicate the predominance of global stabilisation strategies over segmental control. No significant associations were found between temperament and most of the analysed postural parameters, suggesting that temperament does not directly determine global postural organisation. Significant differences between temperament types were identified only for the pelvic torsion DL–DR parameter. Higher values of pelvic torsion DL–DR were observed, particularly in adolescents with sanguine and melancholic temperaments, which may reflect different strategies of neuromuscular control and postural regulation related to functional pelvic alignment. The findings indicate that temperament may play a modulatory role in selected mechanisms responsible for lumbopelvic complex control, especially pelvic torsion. Incorporating temperamental characteristics into postural diagnostics may support the development of more individualised prevention and rehabilitation programmes aimed at improving core stabilisation, motor control and functional control of the lumbopelvic complex in adolescents.

## Figures and Tables

**Table 1:** Basic descriptive statistics of the studied quantitative variables.

Variable	*M*	*Me*	*SD*	*Sk.*	*Kurt.*	*Min.*	*Max.*	*W*	*p*
Height	172.52	172.00	9.15	0.02	−0.75	151.00	196.00	0.98	0.003
Mass	64.85	62.00	13.99	0.90	0.66	40.10	115.20	0.95	<0.001
BMI	21.69	20.80	3.86	1.13	1.16	14.60	35.70	0.92	<0.001
FATP	21.22	21.20	7.19	0.38	−0.36	6.80	46.50	0.97	<0.001
FATM	13.98	12.40	6.46	1.18	1.28	3.30	37.50	0.91	<0.001
PMM	48.30	47.10	10.21	0.48	−0.40	30.10	81.60	0.97	<0.001
BONEM	2.57	2.50	0.51	0.42	−0.47	1.60	4.20	0.97	<0.001
FFM	50.87	49.60	10.72	0.48	−0.40	31.70	85.80	0.97	<0.001
TBW	37.23	36.30	7.84	0.48	−0.40	23.20	62.80	0.97	<0.001
BMR	6940	6795	1336	0.56	−0.23	4597	11,447	0.97	<0.001
Phase angle	5.83	5.82	0.71	0.21	−0.27	4.16	8.06	0.99	0.131
Trunk tilt VP–DM [°]	2.13	2.22	2.70	0.01	0.08	−4.57	9.97	1.00	0.579
Vertical deviation VP–DM [°]	0.01	0.00	1.28	0.12	0.24	−3.65	4.39	1.00	0.828
Vertical deviation VP–DM [mm]	0.08	0.00	10.11	0.18	0.38	−28.50	37.50	1.00	0.524
Pelvic obliquity DL–DR [°]	−0.07	0.00	3.09	0.25	0.66	−9.46	10.30	0.98	<0.001
Pelvic obliquity DL–DR [mm]	−0.22	0.00	5.09	0.08	0.71	−15.00	18.00	0.97	<0.001
Pelvic torsion DL–DR [°]	0.87	1.32	2.69	−0.27	0.05	−7.86	7.90	0.99	0.056
Pelvic tilt (dimples) [°]	20.07	20.17	7.00	−0.42	0.86	−6.61	43.93	0.99	0.006
Kyphosis angle [°]	43.68	43.07	9.64	−0.13	−0.23	18.93	69.67	0.99	0.160
Lordosis angle [°]	35.89	35.16	10.31	0.14	0.10	6.28	65.80	1.00	0.798
Surface rotation (rms) [°]	3.68	3.34	1.73	1.05	1.43	0.67	11.13	0.94	<0.001
Trunk torsion [°]	1.73	1.51	5.12	0.12	1.76	−17.51	21.43	0.98	<0.001
Lateral deviation VP–DM (rms) [mm]	4.83	4.35	2.50	1.37	3.41	1.01	18.24	0.91	<0.001

Correlations between body posture category and temperament type were analysed using the chi-square test. No statistically significant association was found; χ^2^(3) = 5.49, *p* = 0.139, V = 0.14. The results are presented in Table 2.

**Table 3:** Percentage of temperament types in normal and abnormal posture types among students.

	Melancholic		Choleric		Sanguine		Phlegmatic		
Variable	*N*	%	*N*	**%**	*N*	%	*N*	%	Total
Lordosis									
Reduced	44	39.6%	9	8.1%	28	25.2%	30	27.0%	111
Normal	37	41.6%	7	7.9%	24	27.0%	21	23.6%	89
Increased	26	35.6%	6	8.2%	17	23.3%	24	32.9%	73
Kyphosis									
Reduced	52	42.6%	12	9.8%	28	23.0%	30	24.6%	122
Normal	44	37.0%	6	5.0%	34	28.6%	35	29.4%	119
Increased	11	34.4%	4	12.5%	7	21.9%	10	31.3%	32

**Table 4:** Differences in morphometric parameters among four temperament type groups in 16–17-year-old students.

	Melancholic (*n* = 107)		Choleric (*n* = 22)		Sanguine (*n* = 69)		Phlegmatic (*n* = 75)			
Variable	*M*	*SD*	*M*	*SD*	*M*	*SD*	*M*	*SD*	*H*	*p*
BMI	21.76	3.57	21.20	3.69	21.80	3.88	21.64	4.32	1.03	0.795
FATP	21.32	7.32	21.37	7.32	21.06	7.22	21.19	7.09	0.05	0.997
FATM	13.96	6.36	13.74	6.04	14.02	6.56	14.06	6.76	0.05	0.997
PMM	47.85	9.42	46.82	9.41	49.27	10.39	48.49	11.41	1.17	0.761
BONEM	2.54	0.46	2.49	0.47	2.61	0.52	2.58	0.57	1.16	0.763
FFM	50.39	9.88	49.31	9.88	51.88	10.90	51.07	11.98	1.16	0.762
TBW	36.89	7.24	36.05	7.19	37.97	7.98	37.39	8.77	1.21	0.751
BMR	6903.51	1226.90	6715.88	1189.03	7031.71	1369.45	6975.11	1500.22	0.87	0.833
Phase angle	5.86	0.68	5.74	0.54	5.82	0.75	5.82	0.77	0.35	0.951
Trunk tilt VP–DM [°]	2.09	2.61	1.73	2.38	2.14	2.98	2.32	2.66	0.47	0.925
Vertical deviation VP–DM [°]	−0.18	1.29	0.42	1.55	0.05	1.24	0.12	1.21	5.91	0.116
Vertical deviation VP–DM [mm]	−1.39	9.94	3.38	12.44	0.33	10.00	0.98	9.53	5.76	0.124
Pelvic obliquity DL–DR [°]	−0.13	3.04	−0.06	2.89	−0.09	3.72	0.04	2.61	0.39	0.943
Pelvic obliquity DL–DR [mm]	−0.38	4.94	−0.14	4.85	−0.35	6.14	0.09	4.33	0.62	0.891
Pelvic torsion DL–DR [°]	1.01	2.74	−0.25	3.00	1.48	2.41	0.42	2.64	9.96	**0.019**
Pelvic tilt (dimples) [°]	20.50	6.55	20.69	8.05	19.66	6.76	19.64	7.59	0.98	0.806
Kyphosis angle [°]	42.62	9.51	43.85	11.75	43.54	9.60	45.28	9.18	2.87	0.412
Lordosis angle [°]	35.99	9.58	36.98	12.11	35.82	9.58	35.48	11.54	0.03	0.999
Surface rotation (rms) [°]	3.62	1.70	3.35	1.40	3.87	1.83	3.68	1.77	1.51	0.680
Trunk torsion [°]	1.92	5.46	0.85	4.49	1.23	4.97	2.18	4.97	2.37	0.499
Lateral deviation VP-DM (rms) [mm]	4.81	2.63	5.05	2.12	5.03	2.62	4.63	2.31	1.22	0.748

bold values indicate statistical significance.

## Data Availability

The data and materials supporting the conclusions of this article can be found within the article.
